# Seaweeds as Preventive Agents for Cardiovascular Diseases: From Nutrients to Functional Foods

**DOI:** 10.3390/md13116838

**Published:** 2015-11-12

**Authors:** Susana M. Cardoso, Olívia R. Pereira, Ana M. L. Seca, Diana C. G. A. Pinto, Artur M. S. Silva

**Affiliations:** 1 Department of Chemistry & QOPNA, University of Aveiro, Aveiro 3810-193, Portugal; E-Mails: anaseca@uac.pt (A.M.L.S.); diana@ua.pt (D.C.G.A.P.); artur.silva@ua.pt (A.M.S.S.); 2 Department of Diagnostic and Therapeutic Technologies, School of Health Sciences, Polytechnic Institute of Bragança, Bragança 5300-121, Portugal; E-Mail: oliviapereira@ipb.pt; 3 Department of Technological Science and Development, University of Azores, Ponta Delgada 9501-801, Portugal

**Keywords:** macroalgae, algae, heart, hypertension, atherosclerosis, minerals, peptides, sulfated polysaccharides, bioactive, functional food

## Abstract

Being naturally enriched in key nutrients and in various health-promoting compounds, seaweeds represent promising candidates for the design of functional foods. Soluble dietary fibers, peptides, phlorotannins, lipids and minerals are macroalgae’s major compounds that can hold potential in high-value food products derived from macroalgae, including those directed to the cardiovascular-health promotion. This manuscript revises available reported data focusing the role of diet supplementation of macroalgae, or extracts enriched in bioactive compounds from macroalgae origin, in targeting modifiable markers of cardiovascular diseases (CVDs), like dyslipidemia, oxidative stress, vascular inflammation, hypertension, hypercoagulability and activation of the sympathetic and renin-angiotensin systems, among others. At last, the review also describes several products that have been formulated with the use of whole macroalgae or extracts, along with their claimed cardiovascular-associated benefits.

## 1. Introduction

### 1.1. Cardiovascular Diseases (CVDs)

CVDs are a group of disorders of the heart and blood vessels, which include coronary heart disease, cerebrovascular disease, peripheral arterial disease, rheumatic heart disease, congenital heart disease and deep vein thrombosis and pulmonary embolism [1]. Overall, CVDs represent the main cause of death worldwide, with an estimated number of 17.5 million in 2012 (*i.e.*, 31% of the global deaths). From those, approximately 6.7 and 7.4 million deaths were due to coronary heart disease and stroke, respectively. These two diseases are projected to remain the leading causes of death in the coming years and, according to the World Health Organization (WHO), about 23.6 million people will die from CVDs in 2030 [2].

Although there are some non-modifiable risk factors for CVDs such as family history, ethnicity and age, most CVDs arise as a consequence of modern-society habits, including an unhealthy diet, physical inactivity, tobacco use and harmful use of alcohol [3]. These behavior risk factors are normally reflected in individuals by a dyslipidemia, raised blood pressure, overweight and obesity. Considered as traditional biomarkers of CVDs diseases, these parameters are normally screened when evaluating the risk of developing CVDs. Still, the recent growing understanding of multiple biological pathways underlying CVDs, including those of inflammation and oxidative stress [4,5], allowed the identification of additional biomarkers that can be useful for a more authentic and reliable estimation of cardiovascular risk, as well as to monitor the efficacy of treatments and to develop new pharmacological tools [6].

For the last two decades, policies and key actions have been developed in order to effectively control the fast growing burden of CVDs worldwide. Programs offering healthy choices affordable and available, stimulating a healthy life style in populations are a main preemptive approach [7]. In this context, dietary interventions such as reduction of salt in the diet, consuming of fruits and vegetables, food enriched in unsaturated fats and potassium, are accepted as strongly associated with a decreased risk for CVDs [8].

### 1.2. Relevant Macroalgae Components in the Area of Functional Food

Seaweeds, *i.e.*, marine macroalgae are presently pointed out as the plant-based food of the future [9], since besides not competing with food crops for the use of arable land and fresh water resources, they are a good supply of key nutrients including carbohydrates, protein and minerals [10,11], as well as a rich source of health-promoting compounds capable of acting on a wide spectrum of disorders and/or diseases [10,12,13]. This latter fact is becoming particularly evident as macroalgae are presently under the spotlight of many investigations. Note also that macroalgae have been consumed as food in East Asia (mostly in Japan, China and Korea) since ancient times and epidemiological studies associating their regular consumption to several health benefits have been reported [14]. Remarkably, the Japanese have the longest life expectancy in the world and the lowest rates of CVDs, and these scores have been partly associated with their different dietary patterns, which include the regular consumption of macroalgae [15,16].

All these facts are pushing the Western culture to increase the interest in the manufacturing and consumption of high-value products derived from macroalgae, with the main aim of taking advantage of their potential health effects. Indeed, the global functional food market, evaluated for the year 2013, was around $168 billion, and forecast to be more than $305.4 billion by 2020 [17], is believed to be one of such exploiting opportunities where direct applications of seaweeds, crude extracts or purified fractions of seaweeds might hold potential.

Soluble dietary fibers, peptides, phlorotannins, carotenoids and minerals are amongst the most promising macroalgae’s compounds in the area of functional foods/nutraceuticals. Major soluble fibers include alginate from brown macroalgae, carrageenan and agar from red macroalgae, which overall can represent up to half of seaweed’s dried weight (DW). This fact renders macroalgae a leading position with regard to fiber content, ahead of the majority of fruits and vegetables [18,19,20]. This is even more notable because human consumption of fiber-enriched products from macroalgae origin are documented to promote heath benefits, including the prevention of colon cancer, type II diabetes, obesity and CVDs [10,21].

Along with the major soluble fibers mentioned above, fucoidans, *i.e.*, sulfated polysaccharides composed of l-fucose and sulfate ester groups, and ulvans (*i.e.*, sulfated polysaccharides mainly composed of glucuronic acid and iduronic acid units together with rhamnose and xylose sulfates) [13] are also central when it comes to the potential applications in the formulations of functional foods, since over the last few years they have been closely associated to numerous health benefits [22,23]. Note that being natural polysaccharides, their structure is of course very dependent on the species of seaweed. Still, the composition and bioactivities of the final product are also affected by other factors like the method of extraction [13,24].

**Figure 1:**
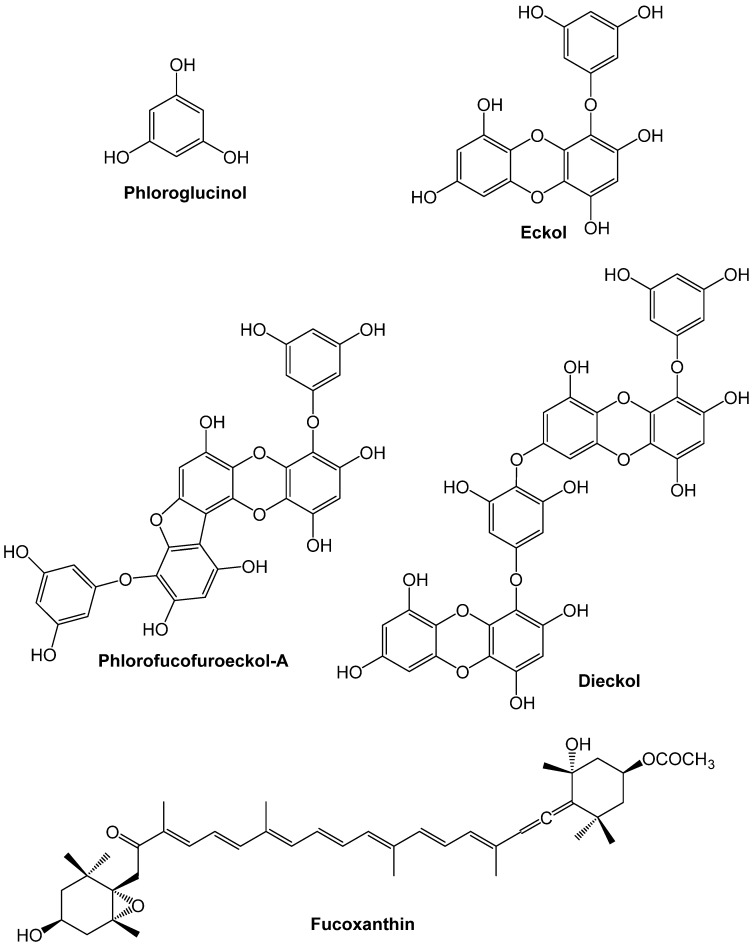
Chemical structures of some phlorotannins (phloroglucinol, eckol, phlorofucofuroeckol-A and dieckol) and fucoxanthin.

Phlorotannins are phenolic compounds exclusive to brown algae that consist of dehydro-oligomers or dehydro-polymers of phloroglucinol (Figure 1), occurring in a wide range of molecular arrangements including variations in the nature of the structural linkages between phloroglucinol units (aryl-aryl or diaryl ether bonds) and the number of hydroxyl groups [25,26]. These phenolic compounds are known to accumulate mainly in the cell cytoplasm in specialized secretion vesicles named physode, representing up to 25% of their DW [26]. Although studies focusing on the characterization and bioactivity of individual phlorotannins are still very limited, several of these compounds have already been isolated and characterized (e.g., phloroglucinol, eckol, phlorofucofuroeckol A and dieckol, see Figure 1) and claimed to provide a wide range of biological activities, including potent antioxidant effects [27].

Macroalgae lipids also have potential applications for exploitation. Seaweeds’ lipids represent only 1%–5% of DW, but almost half of that fraction is polyunsaturated, where omega-3 and omega-6 fatty acids like eicosapentanoic acid and arachidonic acid are abundant [19]. In addition to this, the carotenoid fucoxanthin (Figure 1) has great commercial value since it is accepted to be helpful in the treatment of obesity, while also decreasing the risk of other associated diseases [28].

Besides having high proteic content (10%–47% for red and green) [13,14], macroalgae also contains all essential amino acids, which are undoubtedly of importance for health maintenance. In addition to that, macroalgae peptides containing 3–20 amino acid residues are emerging as bioactive constituents, since distinct authors reported their beneficial actions against several disorders like oxidative stress, hypertension, thrombosis and cancer [10,12,29].

Macroalgae also contain an incomparable wealth of minerals (this may vary from 8% to 40% of algae DW) and have therefore been employed as mineral additives to feed and food supplements [30]. Worth noting is, amongst others, their high content in essential minerals namely Na, K, Mg, P, I, Zn and Fe [11,30]. Indeed, most algae contain high levels of Na and K, but their Na/K ratios are usually low [11,30], which is important to compensate the modern Western diets, typically rich in NaCl. Furthermore, many edible macroalgae have higher contents of Mg than terrestrial plants and animals and some of them are particularly enriched in Ca (up to 2 g/100 g DW) and iodine (up to 0.5 g/100 g DW) [30,31].

## 2. Overview of Relevant Biological Pathways Underlying CVDs

Research studies and accumulated clinical evidence indicate that risk factors of CVDs overlap and intertwine, overall contributing to the onset and growth of the disease. For example, obesity contributes to or directly causes most other modifiable risk factors, including dyslipidemia and hypertension [32] and these in turn are closely related to atheroclerosis and thrombosis. Together with behavior factors, the non-modifiable markers contribute to the onset and exacerbation of CVDs through a cascade of mechanisms including vascular inflammation, oxidative stress, hypercoagulability and activation of the sympathetic and renin-angiotensin systems [4,33]. Attending to the huge complexity of events underlying CVDs, this review will mainly address lipid-metabolic changes and hypertension biomarkers, along with their major complications.

Dyslipidemia is as a major risk factor for CVDs, being in general characterized by an elevated fasting and postprandial concentration of total triglycerides (TG) and of free fatty acids, in combination with the preponderance of low-density lipoproteins cholesterol (LDL-C) along with low levels of high-density lipoproteins cholesterol (HDL-C) and of apolipoprotein B (apo B) plasmatic concentrations [5]. Note that HDL-C exerts cardioprotective effects through the uptake of cholesterol from peripheral tissues and arterial wall to the liver and by preventing LDL oxidation. In opposition, hypertriglyceridemia leads to an incremented formation of small dense LDL, causing the delay of their metabolization and consequent atherogenicity [34]. The latter, in combination with endothelial damage, are key events in the most common pathological processes underlying CVDs [35,36].

Along with dyslipidemia, hypertension is one of the major, yet controllable, highly significant risk factors for the development of all manifestations of CVDs and the main predictor of stroke. It is noteworthy that this condition can also arise from dyslipidemia, through endothelial dysfunction and the loss of physiological vasomotor activity of endothelium, which ultimately result in increased blood pressure and hypertension [35].

By definition, hypertension is established when the systolic blood pressure (*i.e.*, the maximum blood pressure during contraction of the ventricles) is above 140 mmHg and/or the diastolic blood pressure (*i.e.*, the minimum pressure recorded just prior to the next contraction) is above 90 mmHg [37]. Mechanistic causes of hypertension include the reduction of glomerular filtration rate or increased renal tubular reabsorption of salt and water, the excessive activation of renin-angiotensin-aldosterone and sympathetic nervous systems, increased formation of reactive oxygen species (ROS), the vasoconstrictor peptide endothelin and of inflammatory cytokines and decreased synthesis of endothelial nitric oxide. Other important factors for hypertension include an excessive weight gain and dietary factors [38,39].

The intake of minerals and, in particular that of sodium/potassium (Na/K) ratio is believed to be vital in blood pressure control. It is known that the dietary NaCl ingestion increases arterial constriction and peripheral vascular resistance, thus elevating the blood pressure [40]. In opposition, it is well established that K intake leads to the decrement of blood pressure and thus prevents hypertension-associated complications [41]. In this way, WHO recommends a K intake of at least 3.51 g/day and less than 2 g Na/day (equivalent to 5 g salt/day) in adults [41,42]. Besides Na and K, magnesium has also been proven to prevent high blood pressure and metabolic syndrome [43]. Additionally, its deficiency has been suggested to play a role in the onset of type II diabetes and in lipid metabolic changes, thus consequently contributing for atherosclerosis [43,44].

Along with minerals, the renin-angiotensin-aldosterone system has an important function in hypertension, as its activation results in the conversion of angiotensin I to angiotensin II, being the latter a potent vasoconstrictor [45,46]. The clinical use of drugs acting in this system is frequent and includes: (i) inhibitors of angiotensin-converting-enzyme (ACE-I); (ii) angiotensin II receptor blockers (ARBs); and (iii) renin inhibitors. Captopril, cilazapril, enalapril, perindopril, lisinopril and ramipril are authorized ACE-I inhibitor drugs, which competitively block the conversion of angiotensin-I to angiotensin-II [47], while ARBs (e.g., losartan and valsartan) act by competitive antagonism of the angiotensin II receptors [48]. These drugs are used in cases of moderate hypertension and are prescribed alone or in combination with other antihypertensive drugs [46]. Direct renin inhibitors are a novel class of antihypertensive drugs developed to inhibit the renin-angiotensin-aldosterone axis and consequently reduce the angiotensin II concentrations by inhibition of the conversion of angiotensinogen to angiotensin-I [49].

Hypertension and dyslipidemia are also the most common remediable risk factors for atherosclerosis, which is a main cause of the most common pathological process leading to CVDs including myocardial infarction, heart failure and stroke [33,50]. The atherosclerotic plaque is characterized by an increasing lesion grown from a fatty streak to an atheroma, with a fibrous cap. In this process, the passage of lipids into the subendothelial space comes after endothelial damage. It is accepted that endothelium stress can be caused by several factors, including an excess of ROS, or the exposure to toxic agents (e.g., oxidized LDL (oxLDL)), to infectious agents or advanced glycosylated end products (the result of an oxidation reaction with glucose that are found in the blood of diabetics), as well as by hypertension and its proinflammatory effects (smooth muscle lipoxygenase activity and oxide radical formation, among others) [36,50].

The injury of endothelium causes an increase of prothrombotic factors, proinflammatory adhesion molecules (e.g., vascular adhesion molecule-1 (VCAM-1)), proinflammatory cytokines (interleukines 1 and 6 (IL-1 and IL-6, respectively), and tumor necrosis factor alfa (TNF-α)), chemotactic factors (MCP-1), ROS production and increase of leukocyte adhesion. Other important feature is the diminished release of nitric oxide (NO**^•^**) into the arterial wall, which is known to contribute to endothelial homeostasis through diverse mechanisms, including modulation of vascular tone [51,52]. All these events allow amplifying the inflammatory cascade in atherosclerotic lesions, which is characterized by an immunologic and inflammatory infiltrate composed of distinct cells, including monocyte-derived macrophages, smooth muscle cells and T lymphocytes [53]. The development and progression of the lesion depends on the interaction of these cells with the connective tissue, ending on the formation of stable or instead, vulnerable atherosclerotic plaques. The latter are typically characterized by active proteolytic enzymes, including metalloproteinases (MMPs) [45].

Lesions in vulnerable plaques can progress to the thinning of the fibrous cap rendering it susceptible to rupture and thrombosis. In case of rupture, the inflammatory signaling (e.g., T lymphocytes and macrophages) induce the formation of the procoagulant tissue factor, promoting the thrombus formation which is an important complication of atherosclerosis [45,52]. Briefly, local thrombus produces thrombosis-associated mediators (e.g., serotonin, thromboxane A2, thrombin), which cause local and disseminated vasoconstriction. Besides coagulation, platelet activation results in a well-coordinated series of events which includes the trapped platelets to subendothelium, recruitment and activation of additional platelets and the formation of platelet aggregates, which overall contribute for acute atherothrombosis [54].

## 3. Evidences of Protective Effects of Seaweeds with Impact on CVDs

### 3.1. Dyslipidemia

The common side effects of the current synthetic lipid-lowering drugs, *i.e.*, hepatic and/or rhabdomyolysis [55], have increased the tendency to move toward traditional and alternative treatments [56]. In this field, the diet supplementation of seaweeds and/or extracts might have a potential protective role. This is supported by the fact that epidemiological observations show satisfactory results when associating the consumption of seaweeds, medicinal plants and fruits to the prevention of hyperlipidemia in many societies [57]. Having this in mind, the potential effect of distinct macroalgae and/or its isolates in hyperlipidemia control has been largely tested in animal models. Due to the high number of reported studies in this topic, only the most recent ones have been selected for this review.

When supplementing the diets of hypercholesterolemic Wistar rats with 21% of *Himanthalia elongata* or 23% of *Gigartina pistillata*, Villanueva and coworkers [58] showed that *Himanthalia*-treated rats presented a reduction in the plasmatic levels of TG (28%) while increasing those of HDL-C (20%). In turn, the *Gigartina*-supplemented diet produced a significant decrease of 31% in TG, 18% in total cholesterol (TC) and 16% in LDL-C [58]. Identical effect was observed in studies using diets supplemented with tropical green seaweed (5% of dried *Derbesia tenuissima* (DT)). DT did not change total body fat mass but it could decrease the plasmatic levels of TG by 38% and TC by 17% [59].

In addition, the diet supplementation of high-cholesterol/high-fat (HF) Sprague-Dawley rats with 10% freeze dried red seaweed *Gracilaria changii* (*G. changii*) powder for eight weeks significantly lowered the plasmatic TC, LDL-C and TG contents by 40.34%, 35.95% and 30.91% respectively, as compared to the HF-induced rats group although no effect on HDL-C could be observed [60]. Its hypolipidemic action was comparable to statin. As suggested by the authors, one of the potential mechanisms of action explaining the hypolipidemic effects of *G. changii* might be due to its high dietary fiber (61.29%) [60]. Supplementation of *G. changii* to the normal rats showed less significant effect on the plasma TC, HDL-C, LDL-C and TG levels [60]. These differences suggest that this seaweed is probably more effective for hyperlipidemia treatment purposes instead of preventive ones. In addition, brown algae *Ecklonia cava* supplementation dose dependently suppressed TG, TC, and LDL-C concentrations in the serum in both normal and STZ-diabetic mice (supplementation of 5% of seaweed in diabetic mice causes a decrease in serum levels of 72%, 53%, and 78%, respectively) but failed to affect HDL-C concentrations in normal mice [61].

For the last years, there are also distinct works reporting positive effects on lipid metabolism as a result of diet supplementation of extracts obtained from macroalgae. Ruqqia *et al.* [62] showed that amongst the ethanol extracts of 13 seaweed species, those of *Jolyna laminarioides* and *Sargassum binderi* exhibited comparable hypolipidemic potential to common hypolipidemic drugs such as bezafibrate and fenofibrate (through decrement of TC, TG and LDL-C) in diet-induced hyperlipidemic rats and in triton-induced hyperlipidemic rats [62]. The extract from *Melanothamnus afaqhusainii* was also moderately active in lowering the levels of TC, TG and LDL-C in triton-induced hyperlipidemic rats [62]. Liver and cardiac enzymes like lactate dehydrogenase, alkaline phosphatase, aspartate alanine aminotransferase were not adversely affected by administration of these three extracts [62]. 

In addition, the ethanol extract of the brown seaweed *Iyengaria stellate* (10 mg/200 g body weight to rabbits for 30 days) showed an overall decrease in total plasmatic lipid levels, although an increase in the contents of the liver enzymes alkaline phosphatase, glutamic-pyruvic transaminase (SGPT), glutamic oxaloacetic transaminase and γ-transaminase (with exception for SGPT) were also registered [63]. Note that as SGPT is a more specific indicator of liver injury, the overall results gathered by the authors suggest that the intake of *Iyengaria stellate* extract as a hypolipidemic agent should be followed by the monitoring of liver enzymes to ensure liver safety.

Furthermore, Dousip *et al.* [64] investigated the antihyperlipidemic effect of red seaweed *Kappaphycus alvarezii* and brown seaweed *Sargassum polycystum* aqueous extracts, as well as their synergistic effects, in high-cholesterol diet fed rats. The results showed that *Sargassum polycystum* was more effective in decreasing plasma cholesterol (by 37.52%) when compared to *Kappaphycus alvarezii* or with the mixture of the two extracts, though an increase in plasmatic TG levels by 16.66% was also observed. In turn, the *Kappaphycus alvarezii* extract most effectively decreased the TG levels by 40.11% and the mixture of the two extracts further increased HDL-C (by 56.71%). All the three tested conditions (individual extracts or mixture) were able to reduce LDL-C levels when compared to the high-cholesterol group [64]. Overall, hypercholesterolemic rats fed with the mixture of the extracts had the lowest atherogenic index among all groups [64].

Besides the evidences of dietary supplementation of seaweeds and/or crude extracts on lipid metabolism, many authors have also been described positive effects for purified fractions and/or isolated compounds. Amongst them, the majority of the works have been focusing two major groups of compounds, namely seaweeds’ sulfated polysaccharides and lipids. Some recent examples are described below.

Borai *et al.* [65] reported that the oral administration of sulfated polysaccharides (SP) from *Ulva fasciata* to induced-hypercholesterolemic rats for four consecutive weeks did not exert any side effects and, simultaneously, it caused a significant decrement in serum lipid profile by reducing the plasmatic TC, TG, LDL-C and of very low density lipoproteins-cholesterol (VLDL-C). Notably, the scores for *Ulva fasciata* ulvans effects were better than those observed for the reference drug fluvastatin [65]. In addition, Hoang and coworkers [66] reported that two types of SP isolated from the green alga *Monostroma nitidum* showed the ability to reduce cellular lipid concentrations in lipid-loaded hepatocytes, when compared with controls, being this reduction accompanied by a reduced expression of cholesterol synthesis genes and an increment in the expression of genes dictating cholesterol degradation, LDL uptake and peroxisomal β-oxidation.

Another relevant work focusing the effects of ulvans in lipid metabolism has been performed by Hassan *et al.* [67]. These authors described that the intragastric administration of *Ulva lactuca* sulfated polysaccharides to dietary-induced hypercholesterolemic rats could cause a more evident effect in the increment of HDL-C level (by 180%) when compared to that induced by its oral administration [67]. This paves the way for discussion of the effect of the administration mode and how to take advantage of this difference in results.

Along with ulvans, fucoidans have been largely evaluated as antihyperlipidemic agents. Recently, a fucoidan extracted from the brown seaweed *Sargassum henslowianum* and whose structure was established as an α(1→3)-linked l-fucopyranose backbone with sulfate groups mostly present at C-2, C-4 and occasionally at C-3, was tested in an obese mice model. The administration of this fucoidan at a dose of 100 mg/kg P/day to the animals caused a decrement in the plasmatic levels of cholesterol, TG and LDL-C [68]. Unfortunately, definite conclusions on the effective effect of this polysaccharide still requires additional studies on dosage, administration time, in between other parameters.

In addition, Kim *et al.* [69] have recently demonstrated that the dietary supplementation of a commercial fucoidan (Haewon Biotech, Inc., Seoul, Korea) induced a significant decrease on the plasmatic levels of TG, total cholesterol and of LDL-C. As reported by the authors, the beneficial effects of the fucoidan were probably partially associated to the down-regulation of adipogenic transcription factor [69].

Carrageenans, another type of sulfated polysaccharide typically present in red algae, were recently used in a clinical trial study. Patients with ischemic heart disease (IHD) were reported to exhibit a significant effect on lipid profile by a short-term carrageenan supplement [70]. In fact, the prophylactic administration of the carrageenan food supplement in the complex therapy of IHD patients could significantly decrease the plasmatic TC levels by 16.5% and LDL-C by 33.5% as compared with the baseline measurements (background therapy control and experimental groups) [70]. 

Amongst the macroalgae lipids, fucoxanthin and fatty acids are widely mentioned when it comes to dyslipidemia regulation. Notably, the beneficial effects of fucoxanthin on cardiovascular diseases were recently reviewed by Gammone *et al.* [71]. In agreement with distinct reported studies, these authors highlighted that fucoxanthin metabolites (amarouciaxanthin A and fucoxanthinol) are the active *in vivo* forms of fucoxanthin and that their ability in regulating lipids plasmatic levels are mainly due to their antioxidant activity [71]. In addition, fucoxanthin and its metabolite fucoxanthinol are accepted to exhibit extra-metabolic benefic effects, including the regulation of polyunsaturated fatty acids (PUFA) biosynthesis by the promotion of the upregulation of enzymatic activities related to the bioconversion of omega-3 PUFA and omega-6 PUFA to docosahexaenoic acid (DHA) and arachidonic acid (AA), respectively [72,73,74]. Furthermore, it has been demonstrated that both these compounds could induce a decrease in the content of eicosapentaenoic acid (EPA) on cultured rat hepatoma BRL-3A, thus suggesting a down-regulation of metabolic enzymes such as fatty acid desaturase and elongase [75]. In rodents, fed fucoxanthin promotes the synthesis of DHA in the liver [72], resulting in the improvement of the lipid profile since this acid inhibits the synthesis of thromboxane A2 and enhances the production of prostacyclin, a prostaglandin that produces vasodilation and less sticky platelets [76].

As previously mentioned, fatty acids from seaweeds may have itself beneficial properties relevant to cardiovascular disease since they are good sources of the long-chain omega-3 PUFAs EPA and DHA acids [74,77,78,79,80], which in turn promote beneficial effects in serum EPA, TC and TG levels and in LDL-C/HDL-C ratio [77,81,82]. As demonstrated in KK-Ay mouse, DHA and AA levels are significantly increased by the feeding of lipids from *Sargassum horneri* and *Cystoseira hakodatensis*, but not by those obtained from *Undaria pinnatifida* [83]. As suggested by the authors, this difference can be due to the higher fucoxanthin content in the first two seaweeds. The authors have additionally reported that the plasmatic levels of TC, HDL-C and phospholipid of mouse fed with seaweeds lipids were significantly increased while those of hepatic cholesterol and triacylglycerol were decreased as compared with the control group [83]. The increase of serum cholesterol levels by fucoxanthin, in KK-Ay mice, was studied by Beppu *et al.* [84]. The results suggest that fucoxanthin exerts its effects on cholesterol metabolism and in the transport system by down-regulation of the LDL receptor and the class B type 1 scavenger receptor, along with inducing sterol regulatory element binding protein expression [84]. Further studies however are required for clarifying whether the responses to cholesterol metabolism are specific for rodents or extended to Human.

### 3.2. Hypertension

The preventive hypertensive effects of seaweeds have since ever been associated with their richness in dietary fibers and mineral contents and, although reported literature regarding this issue is clearly scarce when compared to that dealing with dyslipidemia, *in vitro* and *in vivo* studies support this association. In addition, recent literature data suggests that bioactive components like fucoxanthin, phlorotannins and peptides can also play a role in seaweeds’ antihypertensive effects.

As mentioned before, inhibition of ACE-I is a well-established approach in the treatment of hypertension and, because of that, many authors have screened the potential of seaweeds in inhibiting this enzyme, either using crude extracts, purified fractions and/or isolated components (see selected examples in Table 1).

Cha *et al.* [85] screened the *in vitro* ACE-I inhibitory activity of methanol and aqueous extracts from twenty-six red Korean algae, obtained at 20 °C or at 70 °C. The authors have found several potential extracts, with IC_50_ values for ACE-I in the range of 12.21–124.69 μg·mL^−1^, being the lowest value found for the aqueous extract of *Lomentaria catenata* at 20 °C. In addition, Jung *et al.* [86] found that among the ethanol extracts of ten Korean seaweeds, including four *Rhodophyta* (*Gelidium amansii*, *Gigartina tenella*, *Porphyra tenera* and *Chondria crassicaulis*), five *Phaeophyta* (*Ecklonia stolonifera*, *Ecklonia cava*, *Hizikia fusiforme*, *Pelvetia siliquosa* and *Undaria pinnatifida*) and one *Chlorophyta* (*Capsosiphon fulvescens*), those of *E. cava*, *E. stolonifera*, *P. siliquosa*, *G. tenella* and *U. pinnatifida* were the most promising in targeting ACE-I, all causing inhibition above 50% at 163.9 μg·mL^−1^. Since several purified fractions and isolated phlorotannins from the extract of *E. stolonifera* also exhibited marked ACE-I inhibitory activity, the authors concluded that phlorotannins, in particular eckol, phlorofucofuroeckol A and dieckol could be partially responsible for the protective activity of the crude extract [86].

In another study, Wisesingh and coworkers [87] also suggested that phlorotannins could be key constituents of extracts when concerning ACE-I targeting. The authors demonstrated that the ethanol extract of *E. cava* was the most active against ACE-I activity when compared to other organic extracts obtained with ethyl acetate, chloroform, hexane or diethyl ether. Besides, they reported that the isolated phlorotannins from this seaweed were active against ACE-I.

Antioxidants, including phlorotannins and fucoxanthin, were also recently shown to be components of *Saccharina japonica* and *Sargassum horneri* oils, either obtained by environmental friendly supercritical-CO_2_ with ethanol as a co-solvent or by conventional extraction using ethanol, hexane or a (1:1, *v*/*v*) mixture of acetone:methanol. All the extracts exhibited moderate-to-high activity against ACE-I, and authors have suggested that this activity could be attributed in part to fucoxanthin, although no direct correlations between its concentration in the extracts and the inhibitory activity were observed. 

Recently, distinct studies applying proteolytic enzymatic digestion to seaweeds has led to detection of a number of renin- or ACE-I-inhibitory bioactive peptide. Examples of potential peptides have been described for red macroalgae (e.g., from *Porphyra columbina*, *Porphyra yezoensis*, and *Palmaria palmata*), as well as for green algae (*Caulerpa microphysa*) and brown algae (*Undaria pinnafita*) (Table 1). As seaweed hydrolysates consist of a complex mixture of constituents and the amino acid sequence of bioactive peptides in ACE-active hydrolysates has not been commonly determined, more experimental data should be gathered in order to allow solid conclusions on structural-active relations. However, according to Suetsuna and coworkers [88], the presence of Tyr residues in dipeptides seems to improve their ability of targeting ACE-I.

**Table 1:** Selected studies reporting inhibitory abilities of seaweeds extracts and isolates on the renin-angiotensin-system.

Seaweed Species	Extraction	Inhibition	Ref
***ACE-I inhibition of extracts***			
Twenty-six red algae	MeOH and Aq Ext 20 °C and 70 °C	Aq Ext 20 °C IC_50_ (µg/mL): *Lomentaria catenata* = 13.78; *Lithophyllum okamurae* = 12.21; MeOH Ext 20 °C IC_50_ (µg/mL): *Ahnfeltiopsis flabelliformis* = 13.84; *Laurencia okamurae* = 106.15; MeOH Ext70 °C: *Bonnemaisonia hamifera*, *Grateloupia filicina*, *Sinkoraena lancifolia*, *Grateloupia lanceolata*, *Gracilaria vermiculophylla* and *L. okamurae* ranging from 25.82 to 124.69	[85]
Ten Korean seaweeds	EtOH Ext	*Ecklonia stolonifera*, *E. cava*, *Pelvetia siliquosa*, *Undaria Pinnatifida* and *Gigartina tenella*: above 50% inhibition of ACE at 163.93 µg/mL	[86]
*Ecklonia cava*	EtOH, EtAc, CHCl_3_, Hex, DE	Best inhibition for EtOH Ext, IC_50_ = 0.96 mg/mL	[87]
***ACE-I inhibition associated with antioxidants***			
*Ecklonia stolonifera*	Purified Phlorotannins	Best inhibition recorded for eckol, dieckol and phlorofucofuroeckol. IC_50_ (μM): eckol = 70.82; phlorofucofuroeckol A = 12.74; dieckol = 34.25	[86]
*Ecklonia cava*	Purified Phlorotannins	IC_50_ (mM): phloroglucinol = 2.57 ± 0.09; eckol = 2.27 ± 0.08; triphlorethol-A = 2.01 ± 0.36; dieckol = 1.47 ± 0.04; eckstolonol = 2.95 ± 0.28	[87]
*Saccharina japonica* (SJ) *Sargassum horneri* (SH)	Supercritic CO_2_ *vs.* Acet: MeOH	IC_50_ (µg/mL): SJ CO_2_ Ext = 0.89 ± 0.07; SJ Acet:MeOH Ext = 1.05 ± 0.14; SH CO_2_ Ext = 0.97 ± 0.11; SH Acet:MeOH Ext = 1.28 ± 0.50;	[89]
***ACE-I or Renin inhibition associated with peptides***			
Porphyra columbina	Enzymatic in thermostatic reactor (A/AF)	ACE-I IC_50_ (g/L): A = 1.2 ± 0.1; AF = 1.7 ± 0.0	[90]
*Porphyra yezoensis*	pH and enzymatic	ACE-I IC_50_ (g/L): 1.6	[91]
*Palmaria palmata*	Papain	Ile-Arg-Leu-Ile-Ile-Val-Leu-Met-Pro-Ile-Leu-Met-Ala Renin inhibitory bioassay: ↓ renin activities by 58.97% (±1.26) at 1 mg/mL.	[92]
*Solieria chordalis* (SC) *Palmaria palmata* (PP)	Chymotrypsin (ChTr) or trysin (Tr)	<10 kDa fractions of SC: hydrolyzed with ChTr (IC_50_ ACE 3.50 mg/mL) or Tr (IC_50_ ACE 20.34 mg/mL); <10 kDa fractions of PP: hydrolyzed with ChTr (ACE IC_50_ 460.05 mg/mL)	[93]
*Caulerpa microphysa*	Pepsin, alcalase, flavourzyme	ACE-I IC_50_ (mg/L): pepsin = 0.20; flavourzyme = 29.74; alcalase = 31.71	[94]
*Undaria pinnafida*	Pepsin	ACE-I IC_50_ (µM): Ala-Ile-Tyr-Lys = 213; Tyr-Lys-Tyr-Tyr = 64.2; Lys-Phe-Tyr-Gly = 90.5; Tyr-Asn-Lys-Leu = 21	[95]
*Undaria pinnatifida*	Aq hot Ext dyalisis, chromatography	ACE-I IC_50_ (µM): Tyr-His = 5.1; Lys-Trp = 10.8; Lys-Tyr = 7.7; Lys-Phe = 28.3; Phe-Tyr = 3.7; Val-Trp = 10.8; Val-Phe = 43.7; Ile-Tyr = 2.7; Ile-Trp = 12.4; Val-Tyr = 11.3	[88]

ACE—angiotensin I converting enzyme; Acet—acetone; Aq—aqueous; Ext—extract; DE—diethyl ether; EtAc—ethyl acetate; EtOH—ethanol; Hex—hexane; MeOH—methanol; ↓ decrement.

Because non-invasive measure of blood pressure in lab animals is a difficult task, this parameter has also not been widely screened when evaluating the beneficial effects of the dietary supplementation of seaweeds or isolates in CVDs-related animal models. Still, some authors described the diet-supplementation of seaweeds and/or seaweed isolates in blood pressure (see resume of selected studies in Table 2).

**Table 2:** Selection of studies that include the assessment of blood pressure or antioxidant, anti-inflammatory or endothelium restoring as a consequence of diet supplementation of seaweeds or isolates, as evaluated *in vivo* models.

Seaweed Species (Extract)	Model Dose	Effects	Ref.
*Ulva ohnoi* (UO) *Derbesia tenuissima* (DT)	High-carbohydrate, HF diet-fed rats 5% for 8 weeks	UO: ↓ total final body fat mass by 24% and sBP by 29 mmHg; ↑ Glc utilization and insulin sensitivity; DT: ↓ TG by 38% and TC by 17%	[59]
*Ulva linza* (UL) *Lessonia trabeculata* (LT)	High-sucrose, HF diet-fed rats|400 mg·kg^−1^ for 8 weeks	UL, LT: ↓ levels of intra-abdominal fat, arterial BP, insulin resistance, TC, TG, SOD; ↓ liver expression levels SOD and GPx and ↑ CAT in control groups and ↓ in algae-fed rats; LT: ↓ GPx activity	[96]
*Gracilaria changii*	HF, HC diet-induced rats|5% and 10% for 8 weeks	5%: ↓ TC (−39.19%), LDL-C (−36.36%), and TG (−25.45%); 10%: ↓ TC, LDL-C and TG content by 40.34%, 35.95% and 30.91%, respectively; lowest AI; 5% and 10%: in plasma = ↓ LipPerox; ↓ AST and ALT levels; in erythrocyte = ↑ SOD, CAT and GSH-Px	[60]
Not detailed	Healthy children from 3 to 6 years diet including seaweed intake using 3-day dietary records	Cross-section study in healthy preschoolers: Girls with higher seaweed intake had significantly lower systolic BP (102.4, 99.2 and 96.9 mmHg for girls with the lowest, middle and highest tertiles of seaweed intake, respectively); seaweed intake was negatively related to dBP in boys and to sBP in girls.	[97]
*Undaria pinnatifida* (UP)	Men/Women with MS|Gr1:1 month (m) 4 g/day UP; Gr2: 1 m 4 g plus 1 m g/day UP (pills)	Randomized double-blinded placebo-controlled trial. Gr2: ↓ systolic BP 10.5 mmHg after a month of 6 g/day seaweed (primarily in subjects with high-normal baseline BP); ↓ waist circumference for women participants (↓ 2.1 cm after 4 g/day and further 1.8 cm after 1 m 6 g/day seaweed). No changes in lipid profile.	[98]
*Undaria pinnatifida* (UP)	19 patients MS|3.3 g in capsules	sBP: ↓ 13 mmHg below the baseline after 4 weeks and 8 mmHg after 8 weeks. dBP: ↓ 9 mmHg after 4 weeks and 8 mmHg after 8 weeks; hypercholesterolemia ↓ 8% by week 4	[99]
***Extracts***			
*Sargassum subrepandum* (MeOH Ext)	Rats with atherogenic diet|100 mg/kg b.wt	↓ TC, TG, LDL-C and ↑ HDL-C; ↓ MDA, NO, leptin, TNF-alpha levels; ↑ adiponectin level;	[100]
*Ulva fasciata* (Ulvans/Aq Ext at 4 °C or 100 °C plus EtOH pp)	HC rats|175 mg/kg for 4 weeks	Both Ext: No side effects; ↓ TC, TG, TG, LDL-C and VLDL-C; ↓ liver NO^•^; ↓ ICAM-1 and VCAM-1; ↑ IL-10; ↓ atherogenic plaques in the aorta more than fluvastatin;	[65]
*Ulva lactuca* (Ulvans/Aq Ext at 100 °C plus EtOH pp)	HC rats|175 mg/kg for 4 weeks	↓TL, TG, TC, LDL-C and VLDL-C; ↑ HDL-C; ↓ AI, creatine kinase and LDH; ↓ liver ALT, AST and ALP activities and serum urea, creatinine and urea/creatinine ratio; ↑ hepatic CAT, GSH-Px; ↑ GSH, Total thiol levels	[67]
Not detailed (Low-MW Commercial alginates)	DOCA salt-induced hypertensive rats|250 or 500 mg/kg for 30 days	↓ sBP; dose-dependent normalization of changes induced by DOCA salt, with the exception of further increasing sodium excretion	[101]
*Gloiopeltis complanata (*Funoran/Aq hot Ext plus various purification steps)	HC, high-sal fed rats|1000 mg/kg for 20 days	↓ sBP; ↓ TC, TG, LDL-C, AI; ↑ urine excretion of sodium, chloride; ↑ urine Na/K ratio	[102]
Not detailed (Seaweed fiber (SF))	Hypertensive Patients|Pills with 0.33 g; 25 min before meals for 4 weeks	↓ mean and sBP; ↑ plasma renin activity; ↓ urinary secretion of Na, K and Na/K ratio	[103]
*Palmaria palmata* (protein hydrolysate and tridecapeptide IRLIIVLMPILMA)	Spontaneously Hypertensive rats|50 mg/kg b.wt	After 24 h ingestion: ↓ 34 mm Hg in sBP; IRLIIVLMPLIMA: ↓ 33 mm Hg	[104]

AI—atherogenic index; Aq—aqueous; ALP—alkaline phosphatase; ALT—alanine aminotransferase; AST—aspartate aminotransferase; BP—blood pressure; CAT—catalase; dBP—diastolic blood pressure; DOCA—deoxycorticosterone acetate; EtOH—etanol; Ext—extract; Glc—glucose; Gr—group; GSH—hepatic reduced glutathione; GSH-Px—glutathione peroxidase; HC—high cholesterol; HDL-C—high density lipoprotein cholesterol; HF—high-fat; LDL-C—low density lipoprotein cholesterol; iCAM—intercelular adhesion molecule-1; LipPerox—lipid peroxidation; MDA—malonaldialdehyde; MeOH—methanol; MS—metabolic syndrome; NO—nitric oxide; pp—precipitation; sBP—sistolic blood pressure SOD—superoxide dismutase; TC—plasma total cholesterol; TG—plasma total triglycerides; VCAM—vascular cell adhesion molecule-1; ↓ decrement; ↑ increase.

When evaluating the response of dietary supplementation of *Ulva ohnoi* and *Derbesia tenuissima* in a rat model of human metabolic syndrome, Kumar *et al.* [59] reported that *Ulva ohnoi* could induce a marked decrease in systolic blood pressure (by 29 mmHg), along with a decrease in the final body fat mass (by 24%) and the improvement of glucose utilization and insulin sensitivity. On the basis of chemical composition of the two algae, the authors have suggested that the better scores of *Ulva ohnoi* with respect to those induced by *Derbesia tenuissima* could be due to its richness in soluble fibers and magnesium. A similar trend on blood pressure were also registered by Ramirez-Huigera *et al.* [96], when testing the effects of diet-suplementation of *Ulva linza* and *Lessonia trabeculata* in rats fed with a hypercaloric diet.

Despite scarce and sometimes inconsistent, the effect of diet supplementation of macroalgae in human blood pressure, either in healthy or in hypertensive scenarios has also been evaluated. The cross-section studies reported by Wada *et al.* [97] in healthy Japanese preschoolers allowed to observe that girls with higher seaweed intake had lower systolic blood pressure while diastolic bold pressure in boys also decreased. Additionally, systolic and diastolic blood pressure in hypertensive elderly Japanese patients have been shown to be significantly decreased after daily doses of 5 g of dried *Undaria pinnatifida* powder for eight weeks [99]. The same trend was registered in a randomized double-blinded placebo-controlled trial with metabolic syndrome patients (also including hypertension) taking daily pill dosages of 6 g of *U. pinnafita* [98].

The benefit of seaweeds’ dietary fiber supplementation in hypertensive rats has also been previously demonstrated by distinct authors. Some authors suggested that their positive effects could be due to retention of dietary Na [102] or to an enhanced intestinal K absorption [101] or to these two effects in simultaneous [103]. It should however be remarked that as the cationic exchange ability of fibers is dependent on factors like the type of fiber and the mineral content of the diet, further information regarding this topic is still required.

Antihypertensive abilities of macroalgaes’ peptides have also been investigated *in vivo* models. Fitzgerald *et al.* [104] showed that the diet supplementation of a tridecapeptide derived from a papain digest of *Palmaria palmata* (IRLIIVLMPILMA, previously shown to be active against renin [92]) could cause a decrease in systolic blood pressure of about 33 mmHg in spontaneously-hypertensive rats. Further simulation of gastric digestion allowed the authors to conclude that the active forms of IRLIIVLMPILMA are probably dipeptides originated along the passage through the gastrointestinal tract. In addition, Suetsuna and coworkers have characterized several di- and tetrapeptides from digests of *Undaria pinnafita* [88,95]*.* Amongst them, the dipetides Tyr-His, Lys-Tyr, Phe-Tyr and Ile-Tyr, as well as the tetrapeptides Ala-Ile-Tyr-Lys, Tyr-Lys-Tyr-Tyr, Lys-Phe-Tyr-Gly and Tyr-Asn-Lys-Leu, were shown to efficiently decrease the blood pressure of spontaneous hypertensive rats through diet supplementation [88,95].

### 3.3. Biological Pathways Underlying Atherosclerotic-Related Events

As previously referred, the formation and progression of atherosclerotic plaques involves a multitude of factors, including oxidative and inflammatory events. In this context, antioxidants and anti-inflammatory sources are of interest in the quest for antiatherogenic activity. Still, it is noteworthy that only a scarce number of research studies have previously monitored these biological pathways when evaluating the potential diet supplementation of macroalgae in atherosclerosis-related models, being those studies major tested with extracts or isolated compounds (included in Table 2). Besides, structure-bioactive studies are almost non-existent. Because of that, this topic demands urgent research.

Phlorotannins are one the seaweeds’ bioactive compounds that could hold a potential in this particular field. In fact, Costa-Mugica and coworkers [105] demonstrated an effective protection of LDL oxidation by phlorotannin-enriched fractions isolated from *Halimeda incrassata*, as measured in two heparin-precipitated LDL models. Moreover, the phlorotannins phloroglucinol, eckol, and dieckol (Figure 1) have been shown to protect against proinflammatory responses in human umbilical vein endothelial cells (HUVEC) and in an animal model of acute inflammation, in response to the endotoxin high-mobility group protein B1 (HMGB1) [106]. Phlorotannins inhibited lipopolysaccharide (LPS)-induced HMGB1 release, HMGB1-mediated barrier disruption, the expressions of CAMs and the adhesion/transendothelial migration of leukocytes to human endothelial cells. The three phlorotannins could also suppress acetic acid-induced hyperpermeability and carboxymethylcellulose-induced leukocytes migration in the animal model [106]. Notably, this study also highlighted the importance of phlorotannins’ hydroxyl groups regarding the vascular barrier protective effects, since the magnitude of protection on HMGB1-mediated hyperpermeability and monocytes adhesion noticed in HUVEC cells followed the pattern dieckol > eckol > phloroglucinol and were even lowered when the hydroxyl groups of dieckol were replaced by methyl groups. Based on the overall results, the authors hypothesized that the presence of hydrogen bond donors and hydrophilic moieties in phlorotannins may play a central role in its affinity for membrane receptors on human endothelial cells [106].

Besides phlorotannins, fucoxanthin has also been associated to the positive effects of a methanol extract of *Sargassum subrepandum* [100]. In their study, the authors showed that in addition to the improvement of lipid profile, the oral administration of the extract in a dose of 100 mg/kg.wt for four months caused a marked decrease in serum malonaldialdehyde (MDA, *i.e.*, a oxidative stress biomarker) and in the proinflammatory markers leptin and TNF-α levels, with a simultaneous increase in the level of adiponectin, *i.e.*, an adipocyte-specific protein that is thought to act as anti-inflammatory, anti-atherogenic and insulin enhancer [107].

Sulfate polysaccharides, including ulvans, carragenans and fucoidans, are the most exploited compounds when regarding the protective effects on atheroclerotic-plaque formation and progression-related problems. e.g., Hassan and coworkers [67] proved that the oral supplementation of hypercholesterolemic rats with an ulvan purified extract from *Ulva lactuca* could ameliorate the activity of the antioxidant enzymes catalase (CAT), glutathione peroxidase (GSH-Px) and superoxide dismutase (SOD) by 110%, 77% and 23%, respectively, when compared with the hypercholesterolemic control rats. Apart from that, the authors also described beneficial effects on non-enzymatic antioxidants parameters (hepatic reduced glutathione, total thiol and lipid peroxidation), suggesting that the defensive effect of ulvan was in part mediated by protection of liver against free radicals.

The atheropreventive activity of ulvans from *Ulva fasciata* was also recently reported by Borai and coworkers [65]. After the oral administration of crude cold and hot extracts to hypercholesterolemic rats extracts at a dose 175 mg/kg body weight for four weeks, the authors registered an improvement of serum lipid profile and of the endothelial dysfunction. In particular, in comparison to hypercholesterolemic rats, the ulvans-supplemented animals showed a decrease of the NO**^•^** liver levels of 38.95% and 69.46% (for cold and hot extracts, respectively), while this effect was of 58.19% for the reference drug (fluvastatin). Both extracts also caused a significant decrease in the levels of the soluble adhesion molecules ICAM-1 and VCAM-1, along with an increase in the amounts of the anti-inflammatory marker IL-10 (about 30%, 18% and 34% compared to control, for cold, hot ulvan-enriched extracts and fluvastatin, respectively). In addition, morphometric measurements of atherosclerotic lesion in untreated/treated rats showed that the oral supplementation of *U. fasciata* extracts induced a reduction in the medial cross-section area of the aorta (19.50% and 26.46%, respectively). This result was also consistent with histological data, which demonstrated that in contrast with the untreated rats, those subjected to algae-enriched diet exhibited no oblivious lesions in the aorta, showing thin intimae and no visible swelling, endothelial cells basically intact and without desquamation, no migration of smooth muscle cells to the under-intimae, no proliferation of smooth muscle cells and smooth muscle cells arranged in a regular pattern.

Amongst SP, fucoidans have very promising biomedical/medical applications with regard to atherosclerosis and associated progressive complications. The anti-inflammatory potential of these polysaccharides in hypercholesterolemic rats has been reported by Preetha *et al.* [108], using a hot water-extracted fucoidan from *Sargassum wightii* and a comercial fucoidan from *Fucus vesiculosos*, which overall resulted in almost equally effects. The subcutaneous treatment with the algae fucoidans at a dose of 5 mg/kg bwt/day during seven days reduced the increased serum levels of the inflammatory biomarkers TNF-α and C-reactive protein, the NO**^•^** concentrations in plasma and cardiac tissues, as well as the levels of cardiac mRNA iNOS and of COX-2 (two potent proinflammatory enzymes). In addition, the authors also demonstrated the anticoagulant effect of these polysaccharides, as estimated by the levels of plasmatic fibrinogen, a central protein in the formation of blood clots.

At this point, one should remark that fucoidans and in general SP have been proved to exert potent antithrombotic effects. Indeed, these polysaccharides share common structural features with heparin, *i.e.*, a highly sulfated glycosaminoglycan that is widely used in clinical practice as an injectable anticoagulant. The anticoagulant activities of SP, along with their inhibition of platelet aggregation and thrombolytic activities has been the focus of many investigations and have been recently revised by distinct authors [22,109,110], which also highlight the high molecular weight and the high sulfation level (particularly with substitution at C-2 or C-2 and C-3 positions of fucose) as positive structural factors affecting these properties [22,111]. Still, reported data focusing these benefits do not take into account bioacessibility and bioavailability issues, as SP *in vivo* models have been administered subcutaneously (as for the above mentioned work of Preetha *et al.* [108]) or, in the majority of cases, as an intravenous injection. In this context, although there are a growing body of evidences that fucoidans and other SP can be partly absorbed [112] or suffer favorable changes by intestinal microbiota [113,114], these topics are not elucidated, hampering the understanding if SP exert effective antithrombotic activities when orally ingested.

## 4. Functional Food with Macroalgae for Promoting Cardiovascular Health

Although there is no consensual definition for the term “functional food” worldwide, this is vastly accepted for foods and food components that have been demonstrated to provide specific health benefits beyond the basic nutrition [19,115]. The design of functional foods is hence undoubtedly associated to the concept of preventing diseases and/or improving optimal health of consumers, besides the basic nutrient needs.

Given the evidence of the beneficial health effects of seaweeds and/or isolates of macroalgae origin, there is a strong case for their inclusion in regular foods (food and beverages), in order to take advantages of their benefits [115,116,117]. It is expected that the combined efforts of industry and research in this field will result in the coming years in a high number of new functional food products reaching to the market, including those intended to promote cardiovascular health.

The development of functional foods with seaweeds for cardiovascular-health promotion have been particularly tested in meat-based products [116,117,118] (Table 3) and patents have also been registered [119]. In these products, it is important to improve the fatty acid composition and the content of functional ingredients, while reducing contents of cholesterol, fat and salt [117,120]. Distinct authors have reported that nutritional values of meat products can be significantly improved by the incorporation of whole seaweeds, without hampering quality and sensory properties [118,121,122,123,124,125,126]. Besides, a remarkable work has been done by Schultz-Moreira *et al.*, since along with describing the enhanced nutritional value of restructured meat when fortified with seaweeds, they also evaluated distinct parameters (e.g., lipid profile, antioxidant enzymes and arylesterase) with impact on cardiovascular system [127,128,129,130,131,132], as demonstrated in hypertensive rats. In addition, Lim *et al.* [133] also showed that chicken and pork patties fortified with *Laminaria japonica* could improve postprandial plasma glucose and lipids profiles in borderline-hyperlipidemic adults.

**Table 3:** Selected studies reporting CVDs-related parameters in foods/beverages using macroalgae or isolates as ingredients.

Product	Seaweed Species	Relevant Results	Ref
***Meat-based products***			
Gel/emulsion meat systems	*Himanthalia elongate* (HE), *Undaria pinnatifida* (UP), *Porphyra umbilicalis* (PU) at 2.5% or 5%	↑ water- and fat-binding properties except in the case of PU at 2.5%.	[121]
Gel/emulsion meat systems	*Himanthalia elongate* (HE), *Undaria pinnatifida* (UP), *Porphyra umbilicalis* (PU) at 5.6%	All: ↑ n-3 PUFA and ↓ n-6/n-3 PUFA ratio; ↓ Na and ↑ K, Ca, Mg, Mn, antioxidants ↓ TI by for PU and UP	[122]
Low-fat frankfurters	*Himanthalia elongata* (HE) at 5.5% (algae plus 50% substitution of animal fat by olive oil)	Effect of HE add: little effect on lipid and amino acid profiles but ↑ dietary fiber content and Ca and ↓ Na/K ratios	[123]
Restructured meats	*Himanthalia elongata* at 5%	Effects in hypercholesterolemic rats: ↓ TC; ↑ expression CYP7A1 and Cu, Zn-SOD; ↓ expression CAT, Mn-SOD and GPx;	[127]
Restructured meats	*Undaria pinnatifida* (UP), *Porphyra umbilicalis* (PU) at 5%	Effects in hypercholesterolemic rats: PU = ↓ TC; ↑ expression Mn-SOD and CAT and AE activity; UP meat mainly had benefits as antioxidant	[131]
Restructured meats	*Undaria pinnatifida* (UP), *Porphyra umbilicalis* (PU) at 5%	UP moderately ameliorated the lipid profile in hypercholesterolemic rats: ↓ TC and VLDL-C	[132]
Restructured meats	*Himanthalia elongata* at 5%	Effects on hypercholesterolemic rats: ↑ AE activity; ↓ VLDL-C, ILDL-C + LDL-C	[130]
Pork/chicken patties	*Laminaria japonica* (LJ) (replacement of 2.25 g pork/chichen by 2.25 g LJ)	↓ increased in postprandial glucose blood levels; ↓ TC and LDL-C	[133]
***Others***			
Bread	Tridecapeptide IRLIIVLMPILMA from *Palmaria palmata* at 4%	Activity against renin IRLIIVLMPILMA maintained after baking process	[134]
Bread	*Ascophyllum nodosum* at 4%	Single blind cross over trial: ↓ in energy intake at a test meal 4 h later; no significant changes in Glc and cholesterol	[135]
Tea	*Ecklonia cava* (EC), *Undaria pinnatifida* (UP), *Hizikia fusiforme* (HF), *Ulva pertusa* (UP)	ACE inhibition IC50 (mg DW/mL): EC = 5.33 ± 0.24, UP = 26.4 ± 1.05, HF = 7.79 ± 0.46; UP = ND	[136]

AE—arylesterase; ACE—angiotensin I converting enzyme; AI—atherogenic index; CAT—catalase; Glc—glucose; IDL-C—intermediate density lipoprotein cholesterol; LDL**-**C—low density lipoprotein cholesterol; PUFA—polynsaturated fatty acids; SOD—superoxide dismutase; TC—plasma total cholesterol; TG—plasma total triglycerides; VLDL-C—very low density lipoprotein cholesterol; ↓ decrement; ↑increase.

Bakery and pasta products are other excellent choices for introducing bioactive ingredients, including algae, since they are greatly consumed worldwide. When testing the incorporation of a renin-inhibitory *Palmaria palmata* protein at 4% in bread, Fitzgerald *et al.* [134] reported that the bioactivity was maintained after the baking process and hence concluded that this peptide could be used for production of bread with enriched renin-inhibitory capacity, while not affecting the texture or sensory properties of the bread to a large degree.

Hall *et al.* [135], in a single blind cross trial also reported that addition of *Ascophyllum nodosum* into bread could significantly reduce energy intake at a subsequent test meal and in the total energy intake in the 24 h period following consumption of the *A. nodosum* enriched bread, though differences in blood glucose and cholesterol were not significant. The authors however suggested that a long-term interventional study should be done in order to establish the real potential of *A. nodosum* enriched bread energy intake as well as in the metabolism of glucose and lipids.

For the last years, development of beverages with seaweeds and/or extracts has also been the focus of distinct investigations and of several patent registrations. Amongst those, Fu and coworkers have patented a beverage containing water-insoluble algal dietary fibers (0.01% to 20%) and citric acid, sugar, fruit juice, plant thickeners and water, which can prevent from distinct diseases, including cardiovascular disorders [137]. Likewise, Kim patented a *Hizikia fusiforme* fortified drink with antihypertensive effects [138], while Korea Bio Polymer Co. Ltd. patented a functional beverage able to ameliorate cardiovascular disease [139]. This latter contained Polymann™ (Korea Bio Polymer Co. Ltd., Seoul, South Korea), a purified form of polymannuronic acid from the kelp *Undaria.*

Besides patents, scientific studies reporting potential effects of functional beverages including distinct macroalge has been reported by Nagai and coworkers [136,140]. Overall, these studies highlighted that drinks formulated with incorporation of macroalgae, in particular with *Ecklonia cava*, could be of benefit not only because of their minerals and phenolics richness, but also because of their ability to target ACE-I.

In between other products, functional salts containing seaweeds components have been studied [141]. Related to this, it is worth noting that there is a commercially available seaweed-derived salt substitute for hypertensive patients (Saloni K, from NMR Pharma, India).

## 5. Conclusions

Seaweeds are a great source of compounds with diverse applications, including in the field of cardiovascular-health. This fact renders macroalgae and crude/purified extracts, a potential for application as ingredients in the formulation of new functional foods in that health area. Indeed, there is evidence that diet supplementation with whole macroalgae or products of macroalgae origin can ameliorate several mechanisms underlying the onset and propagation of CVDs. Still, let us remark that the challenge of using these ingredients in novel foods should not be restricted to the improvement of their nutritional formulations, but instead, efforts should be done in order to test the claimed health benefits of the new products.
